# Synthesis of chiral 3-substituted 3-amino-2-oxindoles through enantioselective catalytic nucleophilic additions to isatin imines

**DOI:** 10.3762/bjoc.14.114

**Published:** 2018-06-06

**Authors:** Hélène Pellissier

**Affiliations:** 1 Aix-Marseille Univ, CNRS, Centrale Marseille, iSm2, Marseille, France

**Keywords:** asymmetric synthesis, chiral 3-amino-2-oxindoles, chirality, isatin imines, nucleophilic addition

## Abstract

This review collects the recent developments in the synthesis of chiral 3-substituted 3-amino-2-oxindoles based on enantioselective catalytic nucleophilic additions to isatin imines published since the beginning of 2015.

## Introduction

Chiral oxindoles represent an important class of products widely present in nature and exhibiting many biological activities. Among them, chiral 3-substituted 3-amino-2-oxindoles constitute privileged candidates in medicinal chemistry [1–8]. Consequently, the development of novel catalytic routes to produce these compounds is highly desired [9–19] with a special mention for organocatalyzed methodologies [10,13]. The simplest method to prepare chiral quaternary 3-amino-2-oxindoles is based on enantioselective catalytic nucleophilic additions to isatin imines. This is not only because of the easy access to isatin imines, but also by the possibility of using a wide range of nucleophiles, thus increasing the structural diversity of the resulting products. The first asymmetric catalytic versions were reported only in the 2010s despite tremendous achievements in catalytic asymmetric imine addition reactions [20–22]. Ever since, a number of catalytic asymmetric nucleophilic additions to isatin imines have been developed, including Mannich reactions, aza-Morita–Baylis–Hillman reactions, Friedel–Crafts reactions, aza-Henry reactions, additions of heteronucleophiles, Strecker reactions, among others. The goal of this review is to update the catalytic asymmetric synthesis of chiral 3-substituted 3-amino-2-oxindoles based on enantioselective nucleophilic additions to isatin imines reported since 2015, since this field was recently reviewed by Chimni et al. [16]. It is divided into six parts, dealing successively with enantioselective Mannich reactions, aza-Morita–Baylis–Hillman reactions, Friedel–Crafts reactions, aza-Henry reactions, domino reactions initiated by nucleophilic additions to isatin imines, and miscellaneous reactions. Most of the reactions depicted in this review have been promoted by a wide variety of chiral organocatalysts but chiral metal catalysts have also proved to be highly efficient for a range of transformations.

## Review

### Enantioselective Mannich reactions

#### Organocatalyzed reactions

Originally, the Mannich reaction is a three-component process occurring between an aldehyde, an amine and a ketone, providing β-amino carbonyl compounds [23–25].

**Scheme 1:**
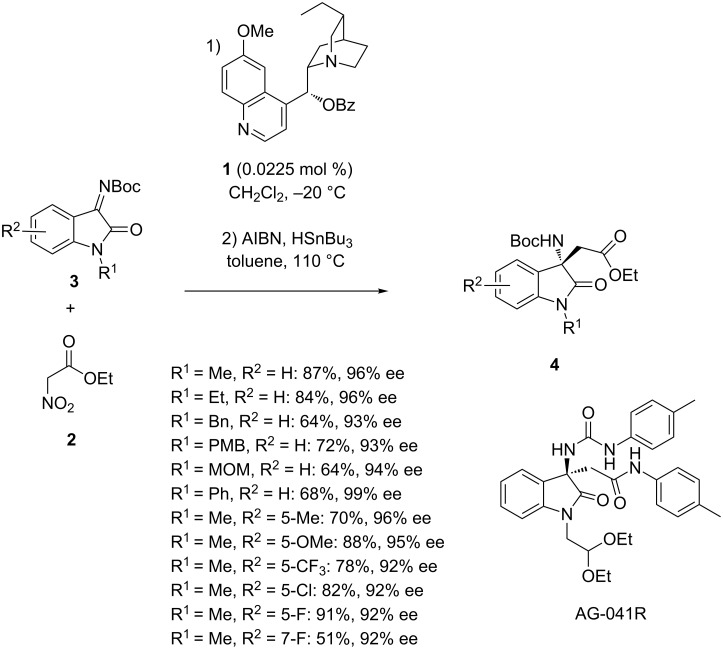
Mannich reaction of *N*-Boc-isatin imines with ethyl nitroacetate (**2**) catalyzed by a cinchona alkaloid followed by denitration and synthesis of AG-041R.

An extensively used two-component variant of this reaction consists in using a preformed imine. Among chiral metal complexes, a wide variety of organocatalysts [26–34] has been used to promote asymmetric Mannich reactions. Among them, cinchona alkaloid **1** was employed in 2015 by Enders et al. at a remarkably low catalyst loading (0.0225 mol %) to promote the enantioselective Mannich reaction of ethyl nitroacetate (**2**) with *N*-Boc-isatin imines **3** [35]. The process afforded, after a subsequent denitration, the corresponding chiral 3-amino-2-oxindoles **4** in moderate to high yields (51–91%) and uniformly high enantioselectivities (92–99% ee), as shown in Scheme 1. Common protecting groups (R^1^), such as methyl, ethyl, 4-methoxybenzyl, methoxymethyl, phenyl and benzyl, were tolerated as well as various substituents (R^2^) on the aromatic ring of the isatins, including electron-donating and electron-withdrawing groups. The lowest yield (51%) was obtained in the reaction of a fluorinated isatin imine (R^1^ = Me, R^2^ = 7-F). The utility of this methodology was demonstrated by its application in the formal synthesis of the anticancer agent AG-041R. Furthermore, several of the formed products were converted into useful intermediates for the synthesis of pyrroloindoline alkaloids and related drugs, such as psychotrimine and (+)-folicanthine.

**Scheme 2:**
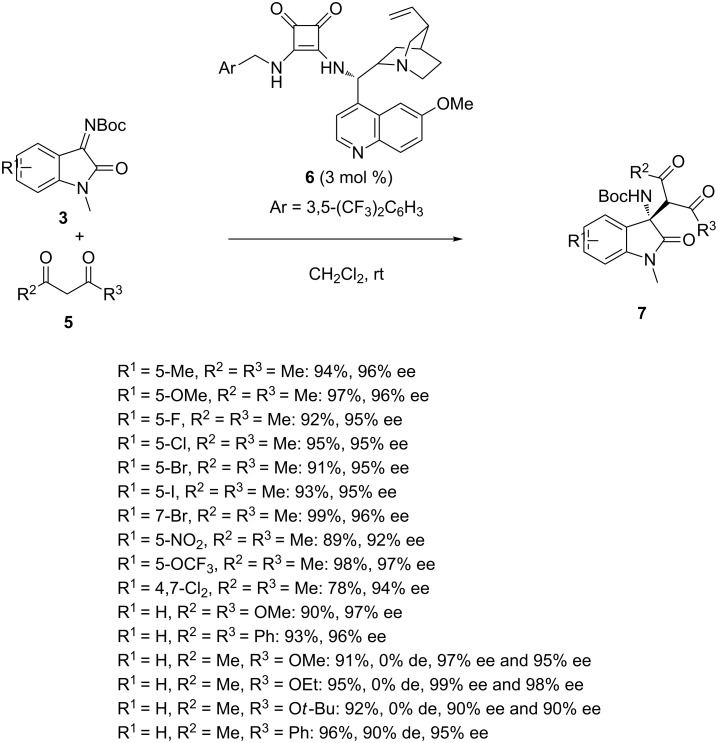
Mannich reaction of *N*-Boc-isatin imines with 1,3-dicarbonyl compounds catalyzed by a cinchona alkaloid-derived squaramide.

Later in 2016, Trivedi et al. reported the synthesis of chiral 3-amino-2-oxindoles through the Mannich reaction of *N-*Boc-isatin imines **3** with 1,3-dicarbonyl compounds **5** performed in the presence of chiral cinchona alkaloid-derived squaramide **6** [36]. A range of chiral 3-amino-2-oxindoles **7** was obtained under mild reaction conditions in high to quantitative yields (78–99%) and uniformly excellent enantioselectivities (90–99% ee) as shown in Scheme 2. In particular, various *N-*Boc-ketimines exhibiting either electron-donating or electron-withdrawing groups at the 5 and 7 positions of the aryl moiety, including halogens such as fluoro, chloro, bromo and iodo groups, reacted smoothly with pentane-2,4-dione (R^2^ = R^3^ = Me) affording the products with both very high yields (89–99%) and enantioselectivities (92–99% ee). A lower yield (78%) albeit combined with a high enantioselectivity (94% ee) was obtained in the reaction of a dihalogenated isatin imine (R^1^ = 4,7-Cl_2_). The scope of the process was also extended to 1,3-dicarbonyl compounds other than symmetrical pentane-2,4-dione, such as 1,3-diphenylpropane-1,3-dione (R^2^ = R^3^ = Ph), that also led by reaction with the unsubstituted isatin imine (R^1^ = H) to the corresponding product in high yield (93%) and enantioselectivity (96% ee). Furthermore, unsymmetrical 1,3-dicarbonyl compounds, such as methyl acetoacetate (R^2^ = Me, R^3^ = OMe), ethyl acetoacetate (R^2^ = Me, R^3^ = OEt) and *tert-*butyl acetoacetate (R^2^ = Me, R^3^ = O*t*-Bu) gave the corresponding Mannich products in high yields (91–95%) and enantioselectivities (90–99% ee), however as a 1:1 mixture of two diastereomers. In contrast, an excellent diastereoselectivity of 90% de was achieved in the reaction of 1-benzoylacetone (R^2^ = Me, R^3^ = Ph) which afforded the corresponding single diastereoisomeric product in 96% yield and 95% ee.

**Scheme 3:**
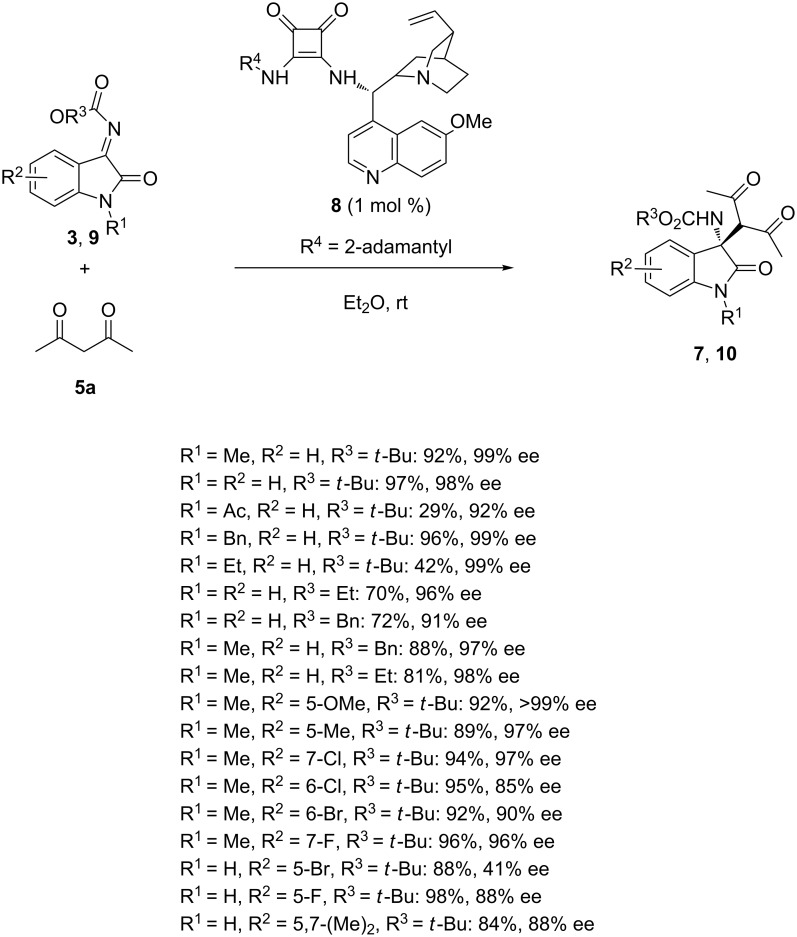
Mannich reaction of *N*-alkoxycarbonylisatin imines with acetylacetone catalyzed by a cinchona alkaloid-derived squaramide.

In 2018, Tanyeli et al. reinvestigated this type of reactions in the presence of related cinchona alkaloid-derived squaramide catalyst **8** [37]. Indeed, the reaction of acetylacetone (**5a**) with various *N*-alkoxycarbonylisatin imines **3** and **9** in the presence of only 1 mol % of catalyst **8** in diethyl ether as solvent afforded at room temperature the corresponding chiral Mannich products **7** and **10** in low to nearly quantitative yields (29–98%) combined with moderate to excellent enantioselectivities (41 to >99% ee) as shown in Scheme 3. The lowest yields (29–42%) were obtained for the ketimines having acetyl and ethyl substituents (R^1^ = Ac or Et) at the amide nitrogen while better yields (70–98%) were generally achieved for other (un)substituted isatin imines (R^1^ = Me, Bn, H). Moreover, a range of electron-withdrawing and electron-donating groups on the aryl moiety (R^2^) as well as different *N-*carbamoyl protecting groups (R^3^ = *t*-Bu, Et, Bn) were compatible, providing generally high enantioselectivities (85 to >99% ee), except for the 5-bromo-substituted derivative (R^2^ = 5-Br) which afforded the corresponding product in only 41% ee. An advantage of this methodology was the use of a very low catalyst loading (1 mol %).

**Scheme 4:**
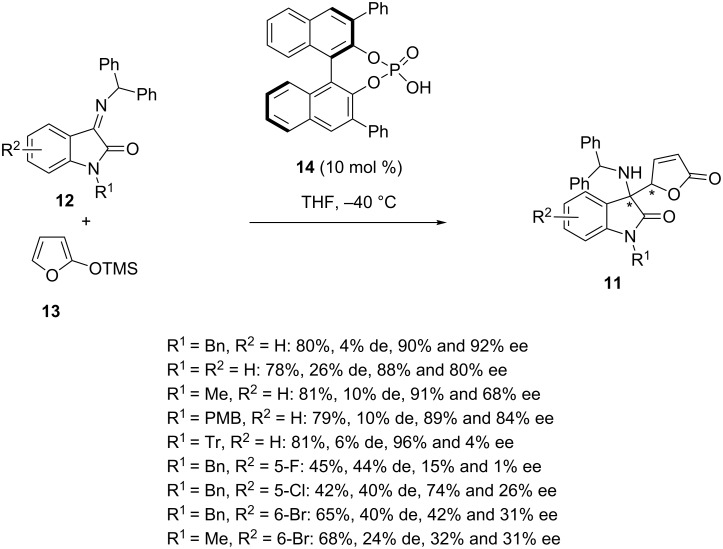
Mannich reaction of isatin-derived benzhydrylketimines with trimethylsiloxyfuran catalyzed by a phosphoric acid.

Earlier in 2016, Silvani and Lesma described the synthesis of chiral 3-amino-2-oxindole butenolides **11** on the basis of an enantioselective organocatalytic Mannich reaction between isatin-derived benzhydrylketimines **12** and trimethylsiloxyfuran **13** [38]. Using 10 mol % of another type of organocatalyst, such as chiral phosphoric acid **14**, the process led at −40 °C in THF to the corresponding butenolides **11** in moderate to good yields (42–81%), low to moderate diastereoselectivities (4–44% de), and low to excellent enantioselectivities (15–96% ee) for the major diastereomers formed (Scheme 4). In general, most of the isatin imines underwent the reaction smoothly with a good level of enantioselectivity (88–96% ee) and good yields (78–81%) while the presence of a halogen substituent on the oxindole moiety (R^2^ = 5-F, 5-Cl, 6-Br) resulted in a lowering of both yields (42–68%) and enantioselectivities (15–74% ee).

**Scheme 5:**
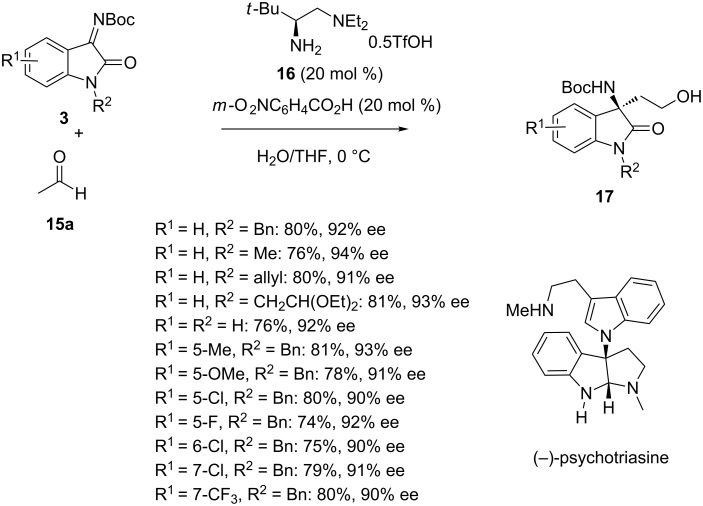
Mannich reaction of *N-*Boc-isatin imines with acetaldehyde catalyzed by a primary amine.

In 2017, Shao et al. investigated the use of simple chiral primary amines to promote the enantioselective Mannich reaction of *N-*Boc-isatin imines **3** with aldehydes, such as acetaldehyde (**15a**) [39]. When these reactions were catalyzed by simple chiral primary amine **16** in aqueous THF at 0 °C, they led to the corresponding chiral Mannich products **17** in good yields (74–81%) and uniformly excellent enantioselectivities (90–94% ee), as shown in Scheme 5. The synthetic utility of this novel methodology was demonstrated through the total synthesis of the natural product (−)-psychotriasine (Scheme 5) and the biologically active compound AG-041R (Scheme 1).

**Scheme 6:**
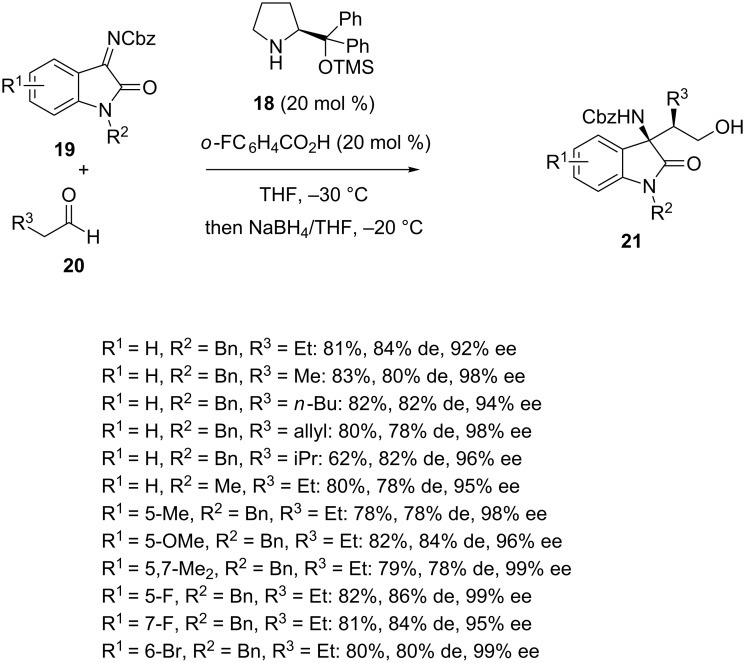
Mannich reaction of *N-*Cbz-isatin imines with aldehydes catalyzed by L-diphenylprolinol trimethylsilyl ether.

By using another type of organocatalyst, such as L-diphenylprolinol trimethylsilyl ether **18**, these authors showed that a range of *N-*Cbz-isatin imines **19** reacted with α-substituted acetaldehydes **20** to give the corresponding chiral Mannich products **21** as major *syn*-diastereomers [39]. The latter were generally obtained with high *syn*/*anti* ratios (89:11 to 93:7) along with good yields (62–83%) and uniformly excellent enantioselectivities (92–99% ee), as shown in Scheme 6.

**Scheme 7:**
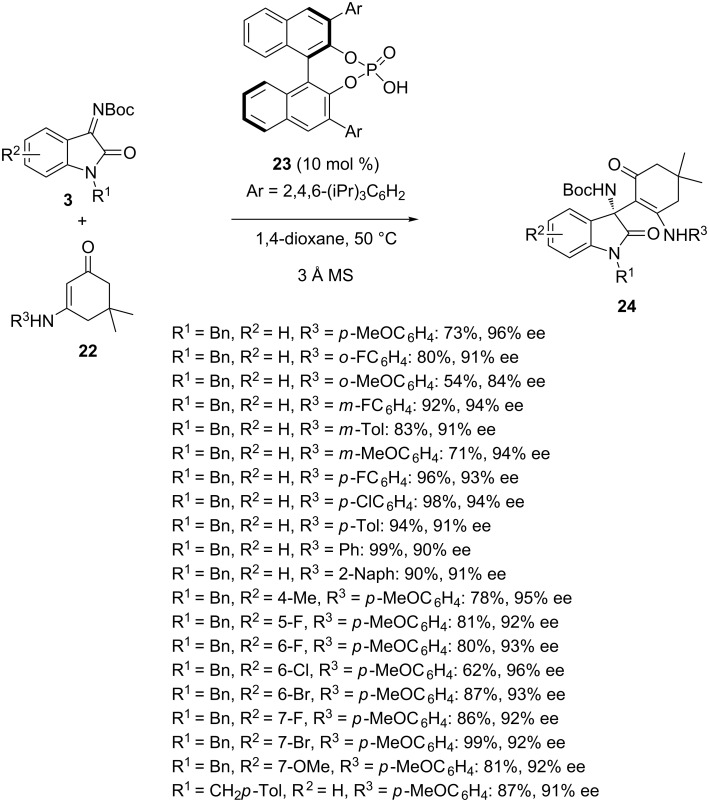
Addition of dimedone-derived enaminones to *N*-Boc-isatin imines catalyzed by a phosphoric acid.

Along with enolates employed as nucleophilic partners in Mannich reactions, related reactions involving enamines have been developed*.* For example in 2018, Lu, Zhang and Shi reported the first catalytic asymmetric construction of the cyclic enaminone-based 3-substituted 3-amino-2-oxindole scaffold with potential bioactivity on the basis of enantioselective additions of cyclic enaminones to *N-*Boc-isatin imines [40]. As shown in Scheme 7, the addition of dimedone-derived enaminones **22** to a variety of *N-*Boc-isatin imines **3** was optimally promoted by chiral phosphoric acid **23** exhibiting a bulky 2,4,6-(iPr)_3_C_6_H_2_ group, which provided at 50 °C in 1,4-dioxane as solvent the corresponding chiral cyclic enaminone-based 3-substituted 3-amino-2-oxindoles **24** in moderate to quantitative yields (54–99%) and good to high enantioselectivities (84–96% ee). The process was compatible with a wide range of *N-*Boc-isatin imines bearing various substituents, the electronic nature and position of which having no obvious effect on the enantioselectivity. Moreover, a variety of dimedone-derived enaminones bearing electronically different substituents at the *ortho*, *meta* and *para-*positions of the aniline moieties was compatible.

**Scheme 8:**
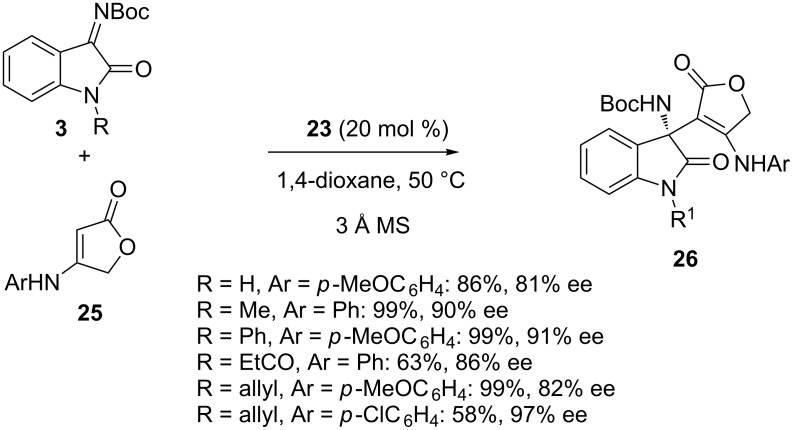
Addition of hydroxyfuran-2-one-derived enaminones to *N*-Boc-isatin imines catalyzed by a phosphoric acid.

The scope of this methodology could be extended to another type of enaminones, such as hydroxyfuran-2-one-derived ones **25**, which afforded by reaction with *N-*Boc-isatin imines **3** the corresponding chiral products **26** in moderate to excellent yields (58–99%) and good to high enantioselectivities (81–97% ee), as illustrated in Scheme 8 [40]. A preliminary evaluation on the cytotoxicity of some selected products revealed that two of them exhibited moderate to strong cytotoxicity to A549, 786-0, ECA109 and BT474 cancer cell lines.

#### Metal-catalyzed reactions

**Scheme 9:**
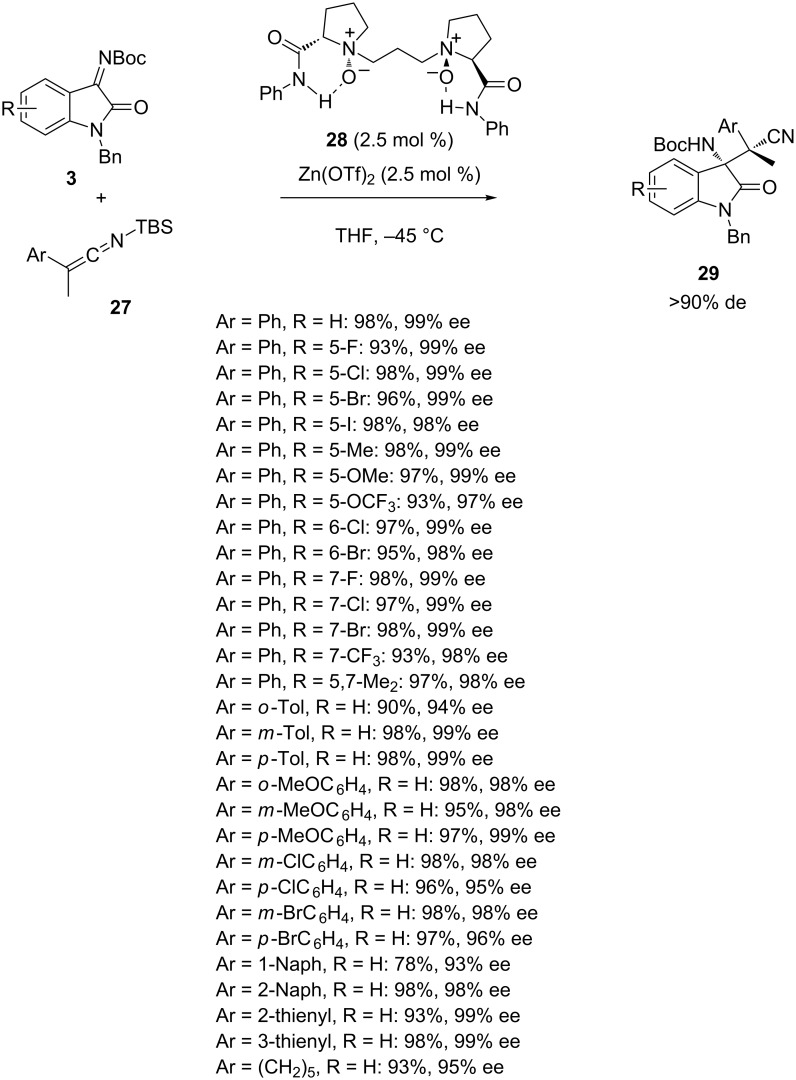
Zinc-catalyzed Mannich reaction of *N-*Boc-isatin imines with silyl ketene imines.

In addition to organocatalysts, chiral metal catalysts [41–47] have been recently applied to promote enantioselective Mannich reactions. As an example, in 2015 Feng et al. reported the enantioselective Mannich reaction of *N-*Boc-isatin imines **3** with silyl ketene imines **27** catalyzed using a combination of Zn(OTf)_2_ and chiral *N,N’-*dioxide ligand **28** [48]. As shown in Scheme 9, this remarkable process afforded a wide range of chiral β-amino nitriles **29** exhibiting two vicinal tetrasubstituted stereocenters as almost single diastereomers (>90% de) in both uniformly excellent yields (90–98%) and enantioselectivities (94–99% ee). In only one case of substrate (Ar = 1-Naph), a lower yield (78%) albeit combined with a comparable very good enantioselectivity (93% ee) was obtained.

**Scheme 10:**
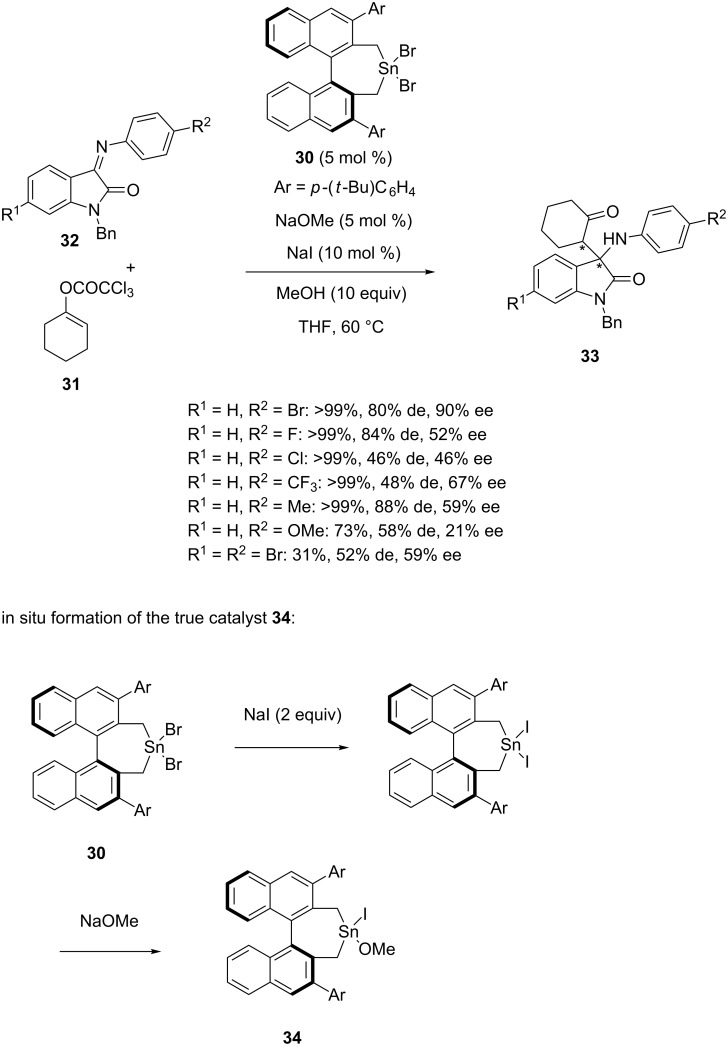
Tin-catalyzed Mannich reaction of *N-*arylisatin imines with an alkenyl trichloroacetate.

The same year, (*S*)-BINOL-derived tin dibromide **30** was employed by Yanagisawa et al. to promote the enantioselective Mannich reaction of alkenyl trichloroacetate **31** with *N-*aryl-isatin imines **32** [49]. The process was performed in the presence of MeONa, NaI and methanol in THF at 60 °C to afford the corresponding chiral 3-alkylated 3-amino-2-oxindoles **33** in most cases in quantitative yields (31 to >99%) combined with moderate to good diastereoselectivities (46–88% de) and enantioselectivities (21–90% ee), as shown in Scheme 10. The best yields were generally achieved in the reaction of isatin imines exhibiting an electron-withdrawing group (R^2^ = Br, F, Cl, CF_3_) at the 6-position of the *N*-phenyl group (>99% yield), while a modest yield (73%) combined with the lowest enantioselectivity (21% ee) was obtained in the reaction of an isatin imine bearing an electron-donating group (R^2^ = OMe). Employing a dibromo-substituted isatin imine (R^1^ = R^2^ = Br) provided the lowest yield (31%). The authors have proposed that the true catalyst of the reaction was a chiral tin iodide methoxide **34** in situ generated from chiral tin bromide **30**, two equivalents of NaI and NaOMe (Scheme 10).

### Enantioselective aza-Morita–Baylis–Hillman reactions

**Scheme 11:**
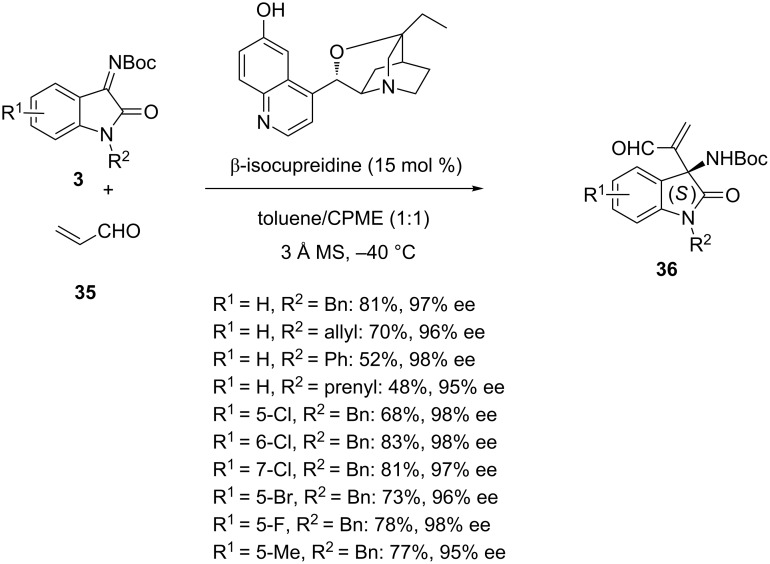
Aza-Morita–Baylis–Hillman reaction of *N-*Boc-isatin imines with acrolein catalyzed by β-isocupreidine.

The Morita–Baylis–Hillman reaction is a carbon–carbon bond-forming reaction occurring between the α-position of an activated alkene and a carbon electrophile such as an aldehyde. Employing a nucleophilic organocatalyst [26], such as a tertiary amine or a phosphine, this simple reaction provides densely functionalized products, such as α-methylene-β-hydroxycarbonyl compounds [50–56]. The aza-variant of this process consists in using an activated imine instead of an aldehyde, thus affording α-methylene-β-aminocarbonyl derivatives. In 2015, Takizawa et al. developed enantioselective aza-Morita–Baylis–Hillman reactions of *N*-Boc-isatin imines **3** with acrolein (**35**) promoted by 15 mol % of β-isocupreidine at −40 °C in a 1:1 mixture of toluene and CPME as solvent [57]. As shown in Scheme 11, the corresponding chiral 3-amino-2-oxindoles **36** were synthesized with uniformly excellent enantioselectivities (95–98% ee) and moderate to good yields (48–83%). Generally, the highest yields (68–83%) were achieved in the reactions of *N-*benzylisatin imines (R^2^ = Bn) while *N-*allyl, *N-*phenyl and *N-*prenyl-substituted ones led to the corresponding products in 48–70% yields.

**Scheme 12:**
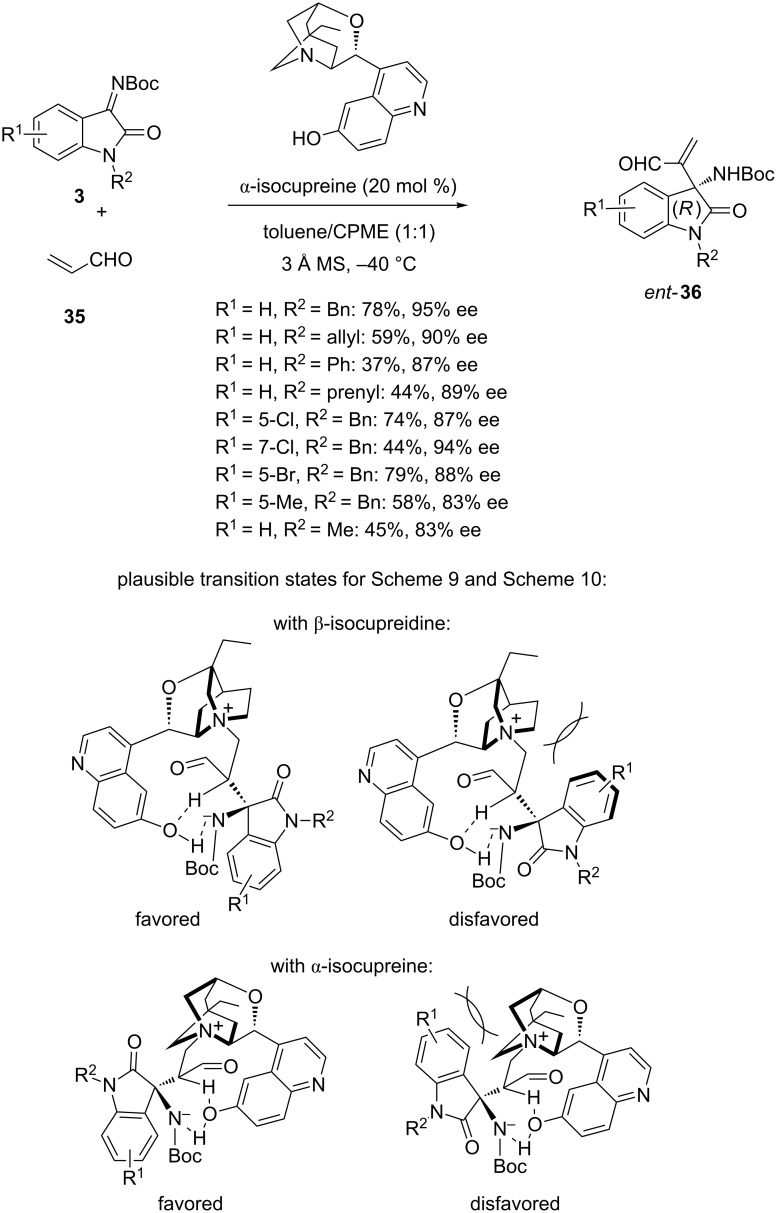
Aza-Morita–Baylis–Hillman reaction of *N-*Boc-isatin imines with acrolein (**35**) catalyzed by α-isocupreine.

Interestingly, the authors found that using 20 mol % of α-isocupreine as organocatalyst instead of 15 mol % of β-isocupreidine (Scheme 11) in this reaction resulted in the formation of the corresponding (*R*)-configured products *ent-***36** with good to high enantioselectivities (83–95% ee) and moderate to good yields (37–79%), as shown in Scheme 12. The stereoselectivity of these processes (Scheme 11 and Scheme 12) can be explained by the favored transition states proposed in Scheme 12 based on the fact that the proton transfer constitutes the rate-determining step in the aza-Morita–Baylis–Hillman reaction [58–60]. Indeed, in these favored transition states, the proton shift mediated by the organocatalyst occurred with the least steric hindrance between the quinuclidine moiety of the catalyst and the aromatic ring of the isatin imine.

**Scheme 13:**
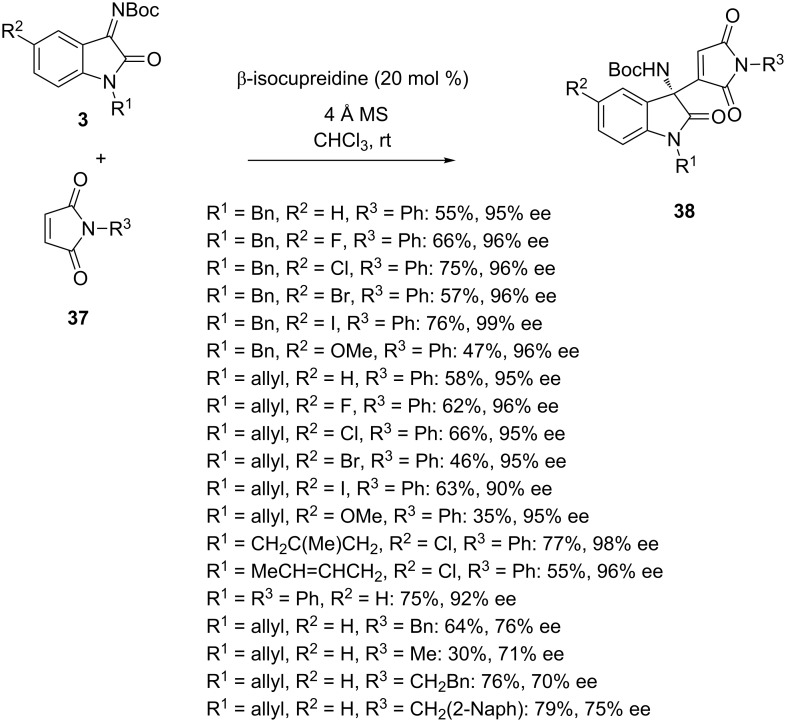
Aza-Morita–Baylis–Hillman reaction of *N-*Boc-isatin imines with maleimides catalyzed by β-isocupreidine.

The same year, Chimni et al. reported organocatalyzed aza-Morita–Baylis–Hillman reactions of *N-*Boc-isatin imines **3** with maleimides **37** using β-isocupreidine as catalyst [61]. It must be noted that maleimides as Morita–Baylis–Hillman donors were challenging in these reactions since they are more usually employed as Michael acceptors. As shown in Scheme 13, a wide variety of chiral 3-amino-2-oxindoles **38** was synthesized in moderate to good yields (30–79%) and enantioselectivities (70–99% ee). The protocol was compatible to differently substituted isatin imines and maleimide derivatives. In particular, uniformly excellent enantioselectivities (90–99% ee) were achieved in the reaction of *N-*phenylmaleimide (R^3^ = Ph) with a range of isatin imines bearing various substituents. However lower levels of enantioselectivity (70–76% ee) were obtained in the reactions of *N-*benzyl (R^3^ = Bn), *N-*methyl (R^3^ = Me), *N-*2-phenylethyl (R^3^ = CH_2_Bn) and *N-*2-(2-naphthyl)ethyl (R^3^ = CH_2_(2-Naph)) maleimides.

**Scheme 14:**
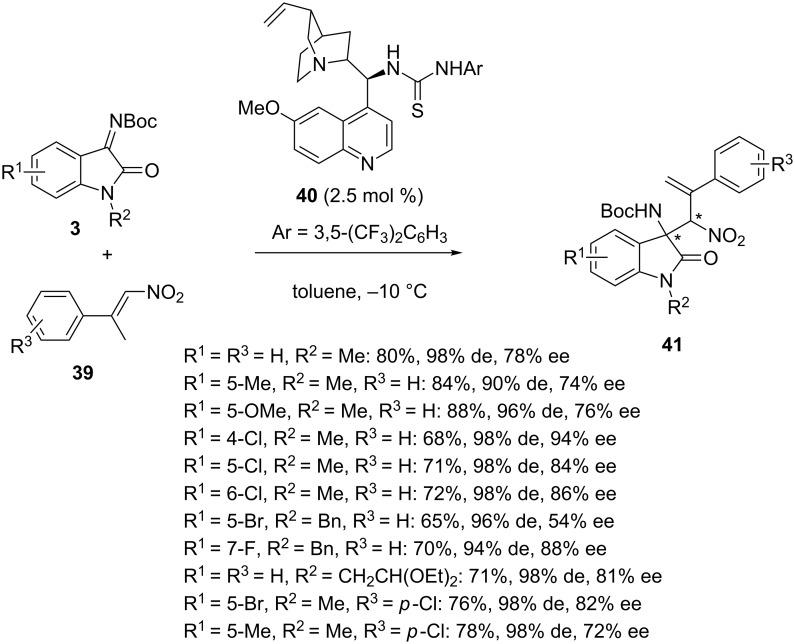
Aza-Morita–Baylis–Hillman reaction of *N-*Boc-isatin imines with nitroolefins catalyzed by a cinchona alkaloid-derived thiourea.

In 2017, Khan et al. reported the first asymmetric organocatalytic synthesis of chiral 3-amino-2-oxindoles based on enantioselective aza-Morita–Baylis–Hillman reaction of *N-*Boc-isatin imines **3** with activated nitroolefins **39** [62]. The best results were achieved by using cinchona alkaloid-derived thiourea catalyst **40** in toluene at −10 °C. Indeed, in the presence of only 2.5 mol % of this quinine-derived organocatalyst, the reaction afforded a range of chiral densely functionalized aza-Morita–Baylis–Hillman products **41** in good to high yields (65–88%), high diastereoselectivities (90–98% de) and moderate to high enantioselectivities (54–94% ee), as shown in Scheme 14. Various electron-donating and electron-withdrawing groups (R^1^) were tolerated on the aryl moiety of the isatin imines, providing good to high yields (68–88%) and enantioselectivities (72–94% ee). On the other hand, the lowest yield (65%) and enantioselectivity (54% ee) were obtained in the reaction of the *N*-benzyl 5-Br substituted substrate (R^1^ = 5-Br, R^2^ = Bn).

### Enantioselective Friedel–Crafts reactions

**Scheme 15:**
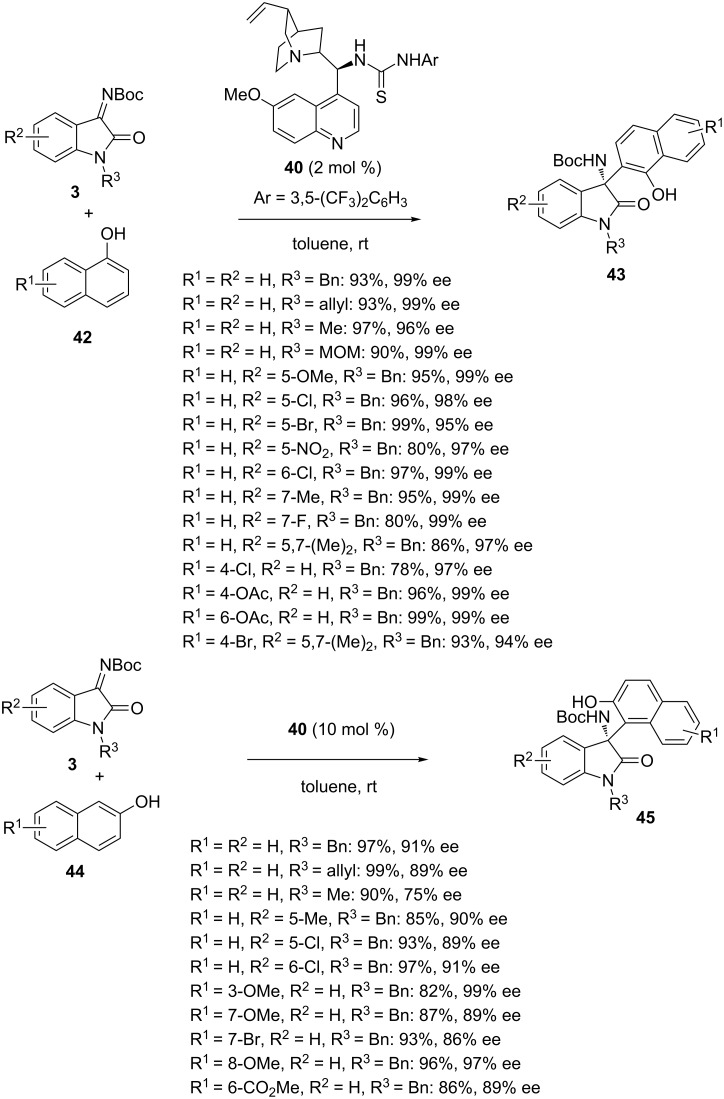
Friedel–Crafts reactions of *N-*Boc-isatin imines with 1 and 2-naphthols catalyzed by a cinchona alkaloid-derived thiourea.

The Friedel–Crafts reaction is widely employed in total synthesis [63–65]. In 2015, Pedro et al*.* reported the first asymmetric Friedel–Crafts reaction of *N*-Boc-isatin imines **3** with naphthols performed in the presence of cinchona alkaloid-derived thiourea **40** [66]. As shown in Scheme 15, the reaction of 1-naphthols **42** promoted by 2 mol % of this bifunctional catalyst in toluene at room temperature led to the corresponding chiral 3-substituted 3-amino-2-oxindoles **43** in good to high yields (78–99%) and uniformly excellent enantioselectivities (94–99% ee) regardless of the electronic character of the aromatic rings of the isatin and 1-naphthol and the position of their substituent (R^1^ and R^2^). Moreover, comparable excellent yields (90–97%) and enantioselectivities (96–99% ee) were achieved with variously *N-*alkyl substituted isatin imines (R^3^ = Bn, allyl, Me, MOM). The scope of this unprecedented methodology was extended to 2-naphthols **44** which required 10 mol % of catalyst loading to provide the optimal results. In these conditions, the reaction with a range of variously substituted *N-*Boc-isatin imines **3** provided the corresponding products **45** in comparable yields (85–99%) and moderate to excellent enantioselectivities (75–99% ee), as illustrated in Scheme 15. A variety of 2-naphthols substituted with electron-donating or electron-withdrawing groups were tolerated, giving good results in most cases. The best enantioselectivity (99% ee) was achieved in the reaction of 3-methoxy-substituted 2-naphthol whereas the lowest one (75% ee) was obtained in the reaction of an *N-*methylisatin imine (R^2^ = H, R^3^ = Me) with unsubstituted 2-naphthol (R^1^ = H). It must be noted that these transformations represented the first highly enantioselective additions of naphthols to ketimines.

**Scheme 16:**
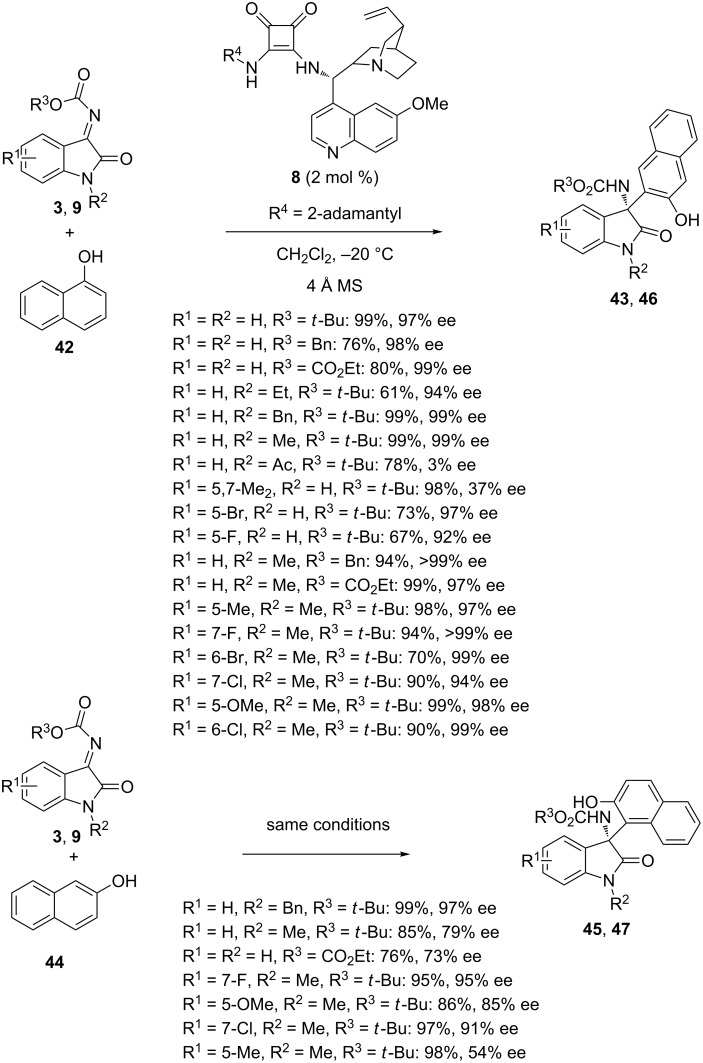
Friedel–Crafts reactions of *N-*alkoxycarbonyl-isatin imines with 1 and 2-naphthols catalyzed by a cinchona alkaloid-derived squaramide.

In 2017, similar reactions were reinvestigated by Tanyeli and Karahan by using the closely related catalyst **8** at −20 °C in dichloromethane as the solvent [67]. The products **43** and **46** arisen from the reaction of 1-naphthols **42** with a range of variously substituted *N-*alkoxycarbonylisatin imines **3** and **9** were obtained in moderate to quantitative yields (61–99%) and excellent enantioselectivities (92 to >99% ee) in most cases. Only two substrates, such as a disubstituted *N*-Boc-isatin imine (R^1^ = 5,7-Me_2_, R^2^ = H) and an *N-*acetylated one (R^1^ = H, R^2^ = Ac), afforded the products in much lower enantioselectivities of 37% and 3% ee, respectively. On the other hand, products **45** and **47** arisen from the reaction of 2-naphthols **44** with *N-*alkoxycarbonylisatin imines **3** and **9** were formed in moderate to excellent enantioselectivities (54–97% ee) combined with good to quantitative yields (76–99%), as shown in Scheme 16. It must be noted that both reactions involving 1 and 2-naphthols employed only 2 mol % of catalyst loading.

**Scheme 17:**
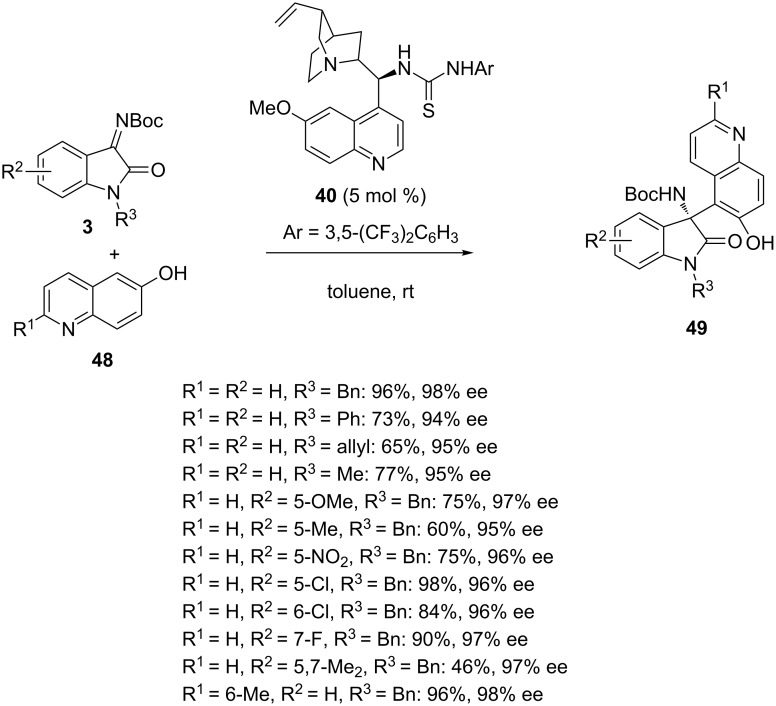
Friedel–Crafts reaction of *N-*Boc-isatin imines with 6-hydroxyquinolines catalyzed by a cinchona alkaloid-derived thiourea.

Very recently, Pedro and Vila reported the enantioselective Friedel–Crafts reaction of *N-*Boc-isatin imines **3** with 6-hydroxyquinolines **48** promoted by the cinchona alkaloid-derived thiourea **40** [68]. The process was performed in toluene at room temperature to give the corresponding chiral 3-amino-2-oxindoles **49** bearing a quinoline moiety in moderate to excellent yields (46–98%) and uniformly excellent enantioselectivities (94–98% ee), as shown in Scheme 17. Comparable results were achieved for *N-*benzyl, *N-*phenyl, *N-*allyl and *N-*methyl-substituted isatin imines (R^3^ = Bn, Ph, allyl, Me). Moreover, various electron-donating or electron-withdrawing groups were tolerated at the 5, 6 or 7-position of the isatin aryl moiety (R^2^). The lowest yield (46%) was obtained in the reaction of a disubstituted ketimine (R^2^ = 5,7-Me_2_). It must be noted that this methodology represented the first enantioselective addition of hydroxyquinolines to imines.

### Enantioselective aza-Henry reactions

**Scheme 18:**
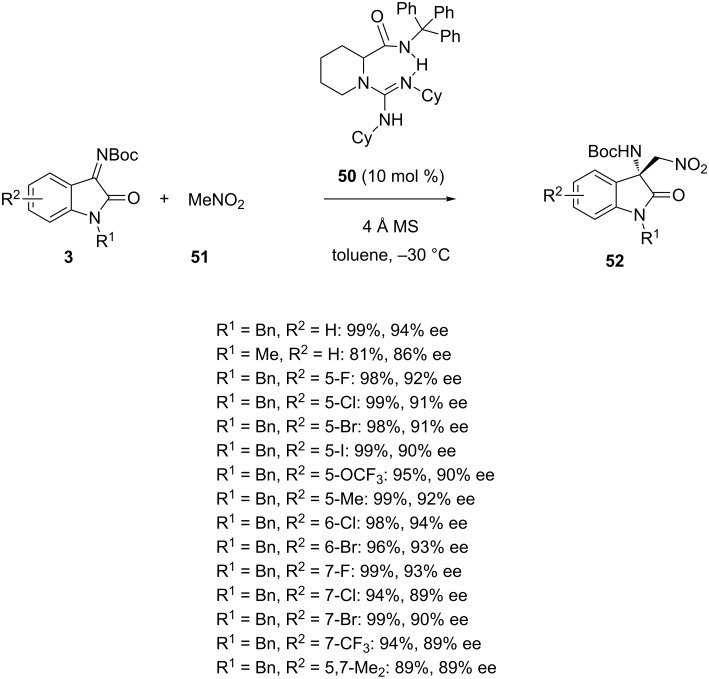
Aza-Henry reaction of *N-*Boc-isatin imines with nitromethane catalyzed by a bifunctional guanidine.

The catalytic asymmetric Henry reaction, also known as catalytic asymmetric nitroaldol reaction, constitutes a useful synthetic methodology towards chiral β-nitro alcohols [69–70]. This reaction and related aza-Henry reactions involving imines have been promoted by both metal catalysts and organocatalysts. While isatins have been widely used as substrates in asymmetric Henry reactions, isatin imines have been less explored as aza-Henry acceptors in enantioselective reactions. Recently, several groups have developed enantioselective aza-Henry reactions of isatin imines catalyzed by either chiral organocatalysts or chiral metal complexes. Among the organocatalysts, chiral bifunctional guanidine **50** was applied by Feng et al. in 2015 to promote the reaction of nitromethane **51** with *N-*Boc-isatin imines **3** (Scheme 18) [71]. The reaction performed at −30 °C using 10 mol % of this catalyst in toluene provided a range of chiral aza-Henry products **52** in high to quantitative yields (81–99%) and high enantioselectivities (86–94% ee). Generally, *N-*benzyl-substituted isatin imines (R^1^ = Bn) provided better yields (89–99%) and enantioselectivities (89–94% ee) than an *N-*methyl-substituted isatin imine (81% yield, 86% ee with R^1^ = Me, R^2^ = H).

### Domino reactions

**Scheme 19:**
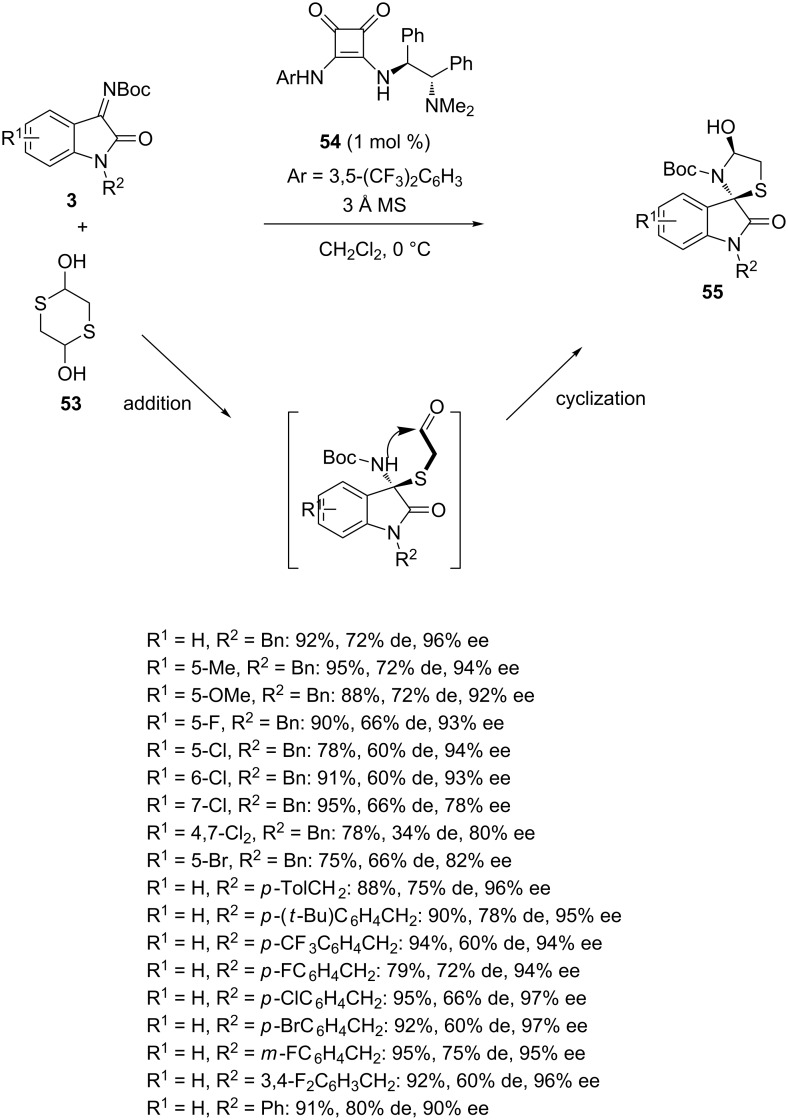
Domino addition/cyclization reaction of *N-*Boc-isatin imines with 1,4-dithiane-2,5-diol (**53**) catalyzed by a tertiary amine-squaramide.

In the last decade, a number of highly enantioselective domino processes [72–74] catalyzed by either chiral organocatalysts [26] or chiral metals have been published [75–77]. In 2016, Li and Li reported an efficient asymmetric formal [3 + 2] annulation reaction between *N-*Boc-isatin imines **3** and 1,4-dithiane-2,5-diol (**53**) as equal equivalent of 2-mercaptoacetaldehyde [78]. The domino reaction catalyzed by chiral tertiary amine-squaramide catalyst **54** began with the addition of 2-mercaptoacetaldehyde to isatin imine **3**, leading to an aldehyde intermediate which subsequently cyclized into the corresponding chiral spirocyclic 3-aminooxindole **55**. As shown in Scheme 19, a range of these products was achieved in good to high yields (75–95%) and enantioselectivities (78–97% ee) combined with low to good diastereoselectivities (34–80% de). The lowest diastereoselectivity (34% de) was obtained in the reaction of a disubstituted substrate (R^1^ = 4,7-Cl_2_), showing that it was sensitive to the substitution of the aryl moiety of the isatin. In fact higher diastereoselectivities (≥60% de) were achieved for all other substrates variously substituted with electron-donating or electron-withdrawing groups (R^1^). In addition to *N-*benzyl-substituted isatin imines (R^2^ = Bn), the reaction conditions could be applied to a range of *N-*substituted benzyl substrates which all provided the products in comparable high yields (75–95%) and enantioselectivities (94–97% ee) along with moderate to good diastereoselectivities (60–78% de). Furthermore, an *N-*phenylisatin imine (R^2^ = Ph, R^1^ = H) was found to smoothly undergo the reaction with excellent results (91% yield, 80% de, 90% ee).

### Miscellaneous reactions

**Scheme 20:**
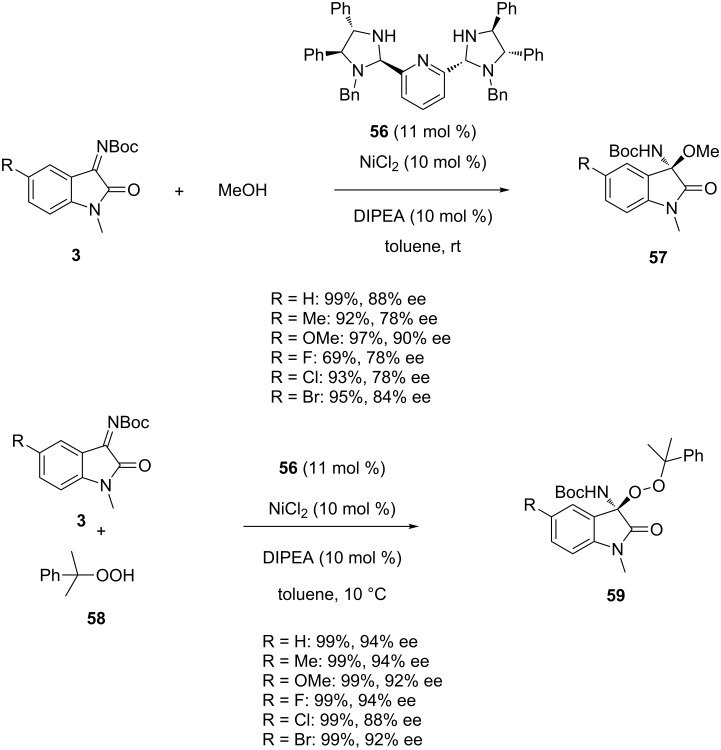
Nickel-catalyzed additions of methanol and cumene hydroperoxide to *N-*Boc-isatin imines.

In 2015, Arai et al*.* applied a chiral metal complex to promote the enantioselective addition of methanol to *N-*Boc-isatin imines **3** [79]. As shown in Scheme 20, the use of a chiral nickel catalyst in situ generated from NiCl_2_ and the chiral bis(imidazolidine)pyridine ligand **56** promoted this reaction in toluene at room temperature in the presence of a base, such as DIPEA, and led to chiral isatin-derived *N,O*-acetals **57** in good to quantitative yields (69–99%) and good to high enantioselectivities (78–90% ee). Notably, generally excellent yields (93–99%) were obtained in the reactions of variously substituted substrates while the lowest yield of 69% was observed for the 5-fluorinated isatin imine (R = F). Comparable reaction conditions were applied to the addition of cumene hydroperoxide **58** to *N-*Boc-isatin imines **3** providing the corresponding chiral oxindoles **59** in even higher yields (99%) and enantioselectivities (88–94% ee), as shown in Scheme 20.

**Scheme 21:**
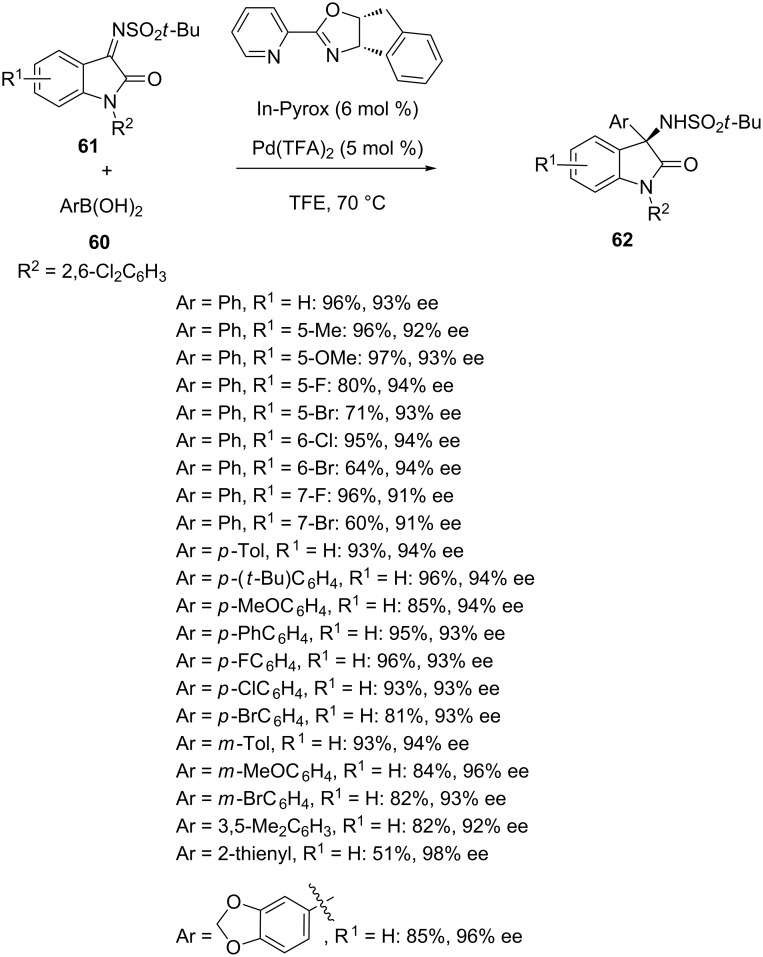
Palladium-catalyzed addition of arylboronic acids to *N-tert*-butylsulfonylisatin imines.

In 2016, Zhang and Yang reported an asymmetric palladium-catalyzed addition of arylboronic acids **60** to sterically hindered *N-tert*-butylsulfonylisatin imines **61** [80]. Among a variety of chiral ligands investigated, including different pyridine-oxazolines, oxazolines and (*R*)-BINAP, the chiral pyridine-oxazoline ligand In-Pyrox was found optimal to provide a wide variety of chiral 3-amino-2-oxindoles **62** in moderate to high yields (51–97%) and uniformly excellent enantioselectivities (91–98% ee). As shown in Scheme 21, the reaction performed at 70 °C in TFE as solvent proceeded well in the presence of various substituents (R^1^) at different positions of the aryl moiety of isatin imines, giving comparable enantioselectivities (91–94% ee). Moreover, the arylboronic acid scope was also found wide and various *para-* as well as *meta*-substituted arylboronic acids reacted smoothly, giving the products in high yields (81–96%) and enantioselectivities (93–96% ee). Good yields (82–85%) and high enantioselectivities (92–96% ee) were also obtained for disubstituted arylboronic acids. Even a heteroaromatic boronic acid (Ar = 2-thienyl) was tolerated, providing the corresponding product in excellent enantioselectivity (98% ee) albeit with moderate yield (51%). However, no reaction occurred with *ortho-*substituted substrates. This process constituted the first example of a palladium(II)-catalyzed addition of arylborons to exocyclic ketimines.

## Conclusion

This review demonstrates that much progress has been achieved in the past three years in the field of asymmetric synthesis of 3-substituted 3-amino-2-oxindoles through enantioselective nucleophilic additions to isatin imines, involving both chiral metal and organocatalysts. Indeed, a variety of novel methodologies, including enantioselective Mannich reactions, aza-Morita–Baylis–Hilman reactions, Friedel–Crafts reactions, aza-Henry reactions, domino reactions, among others have allowed a wide range of these biologically important chiral products to be achieved in generally excellent enantioselectivities and high yields by using different types of metal ligands and organocatalysts. For example, remarkable enantioselectivities of up to 94 to >99% ee have been reported in recent examples of Mannich reactions promoted by organocatalysts, as varied as cinchona*-*alkaloids, squaramides, phosphoric acids, simple primary amines and L-diphenylprolinol trimethylsilyl ether. However, also metal complexes (Zn, Sn) derived from *N,N’*-dioxide and BINOL-derived ligands have been employed affording the products with enantioselectivities of up to 90–99% ee. Moreover, comparable enantioselectivities of up to 99% ee were reported in aza-Morita–Baylis–Hilman reactions organocatalyzed by cinchona-alkaloids, and unprecedented Friedel–Crafts reactions of isatin imines with naphthols and hydroxyquinolines promoted by cinchona*-*alkaloid-derived thioureas and cinchona-alkaloid-derived squaramides. Slightly lower enantioselectivity levels of up to 94% ee were described in aza-Henry reactions performed with bifunctional guanidines. In addition, excellent results (97% ee) were reported in unprecedented domino reactions promoted by squaramides recently. Miscellaneous transformations, including additions of methanol, hydroperoxides and arylboronic acids, were also developed with high enantioselectivities (up to 94% ee) by using chiral nickel and palladium complexes with imidazoline and pyridine-oxazoline ligands while the first additions of enaminones to isatin imines catalyzed by chiral phosphoric acids provided even higher enantioselectivities (up to 97% ee). In spite of these significant advances, the involvement of other types of organocatalysts and metal ligands will have to be investigated in these transformations along with the use of other nucleophiles to even more extend the library of chiral 3-substituted 3-amino-2-oxindoles available for drug discovery.
